# Removal of Seventeen Inches of the Small Intestines, and Recovery of the Patient

**Published:** 1845-06-01

**Authors:** 


Removal of seventeen inches of the small intestine and recovery of the patient.—Dr. Brigham, Superintendent of the State Lunatic

Asylum at Utica, communicates for the April number of the Am. Journal of Medical Sciences, (1845) the case of an insane patient, who, with a pair of large scissors, made "two wounds into her abdomen; one about an inch and a half above the umbilicus, and the other half an inch below it. From the upper opening she took out part of the small intestines, from which she cut off *seventeen inches* in length."

When discovered by Dr. Buttolph, the assistant Physician, one end of the intestine from which the portion was separated was withdrawn into the abdomen. Supposing the case would certainly prove fatal, he simply returned the other end, stitched and dressed the wound carefully, and directed an injection of laudanum. No abdominal inflammation ensued. She was kept very quiet; injections of laudanum, and broth administered. After a few days she took and retained a little food. "*Thirty-three* days after the accident she had a slight discharge from the bowels of hardened faeces, and on the next day a copious one. The wounds had already healed, and she was soon able to walk about. Since that time she has continued to have regular evacuations from the bowels, though there is a tendency to diarrhea. She is able to be about the house, and is as well as she was for several weeks previous to the injury."

